# Case Report: Durable complete response to olaparib in a patient with BRCA2-driven metastatic gastric adenocarcinoma after failure of chemo-immunotherapy

**DOI:** 10.3389/fonc.2026.1769043

**Published:** 2026-04-07

**Authors:** Gökhan Karakaya

**Affiliations:** Akdeniz Saglik Vakfi Yasam Hastanesi, Antalya, Türkiye

**Keywords:** BRCA-2, gastric adenocarcinoma, olaparib, PARP inhibitor, precision oncology

## Abstract

Metastatic gastric adenocarcinoma is associated with poor prognosis and limited treatment options after failure of chemotherapy and immunotherapy. Although BRCA1/2 alterations are well-established predictive biomarkers for PARP inhibitor efficacy in several malignancies, their role in gastric cancer remains incompletely defined. We report a 65-year-old male with metastatic gastric adenocarcinoma who underwent gastrectomy with intraoperatively detected liver metastases. The disease progressed following platinum-based chemotherapy combined with nivolumab and subsequent paclitaxel-ramucirumab therapy. Comprehensive genomic profiling using the Tempus xT assay identified a pathogenic germline BRCA2 frameshift mutation with somatic loss of heterozygosity. Based on this molecular profile, off-label olaparib therapy was initiated. The patient achieved a complete metabolic response on FDG PET-CT, which has been sustained for more than three years under continuous olaparib therapy with acceptable tolerability. This case highlights the importance of molecularly guided treatment strategies and homologous recombination repair deficiency in selected patients with advanced gastric cancer.

## Introduction

Despite advances in systemic therapy, metastatic gastric cancer remains a disease with limited long-term survival (1). Platinum-based chemotherapy combined with immune checkpoint inhibitors constitutes a standard first-line approach for selected patients; however, progression is common, and effective treatment options beyond second-line therapy are scarce (2). Precision oncology approaches based on molecular profiling are increasingly being explored, yet their clinical application in gastric cancer remains limited.

Alterations in homologous recombination repair (HRR) genes, particularly BRCA1 and BRCA2, predict sensitivity to PARP inhibitors in breast, ovarian, pancreatic, and prostate cancers (3, 4). In gastric cancer, however, clinical trials of PARP inhibitors in unselected populations have largely failed to demonstrate meaningful benefit, underscoring the importance of appropriate biomarker selection (5). Herein, we present a case of BRCA2-driven metastatic gastric adenocarcinoma achieving a durable complete response to olaparib after failure of chemo-immunotherapy and anti-angiogenic treatment.

## Case presentation

**Figure 1:**
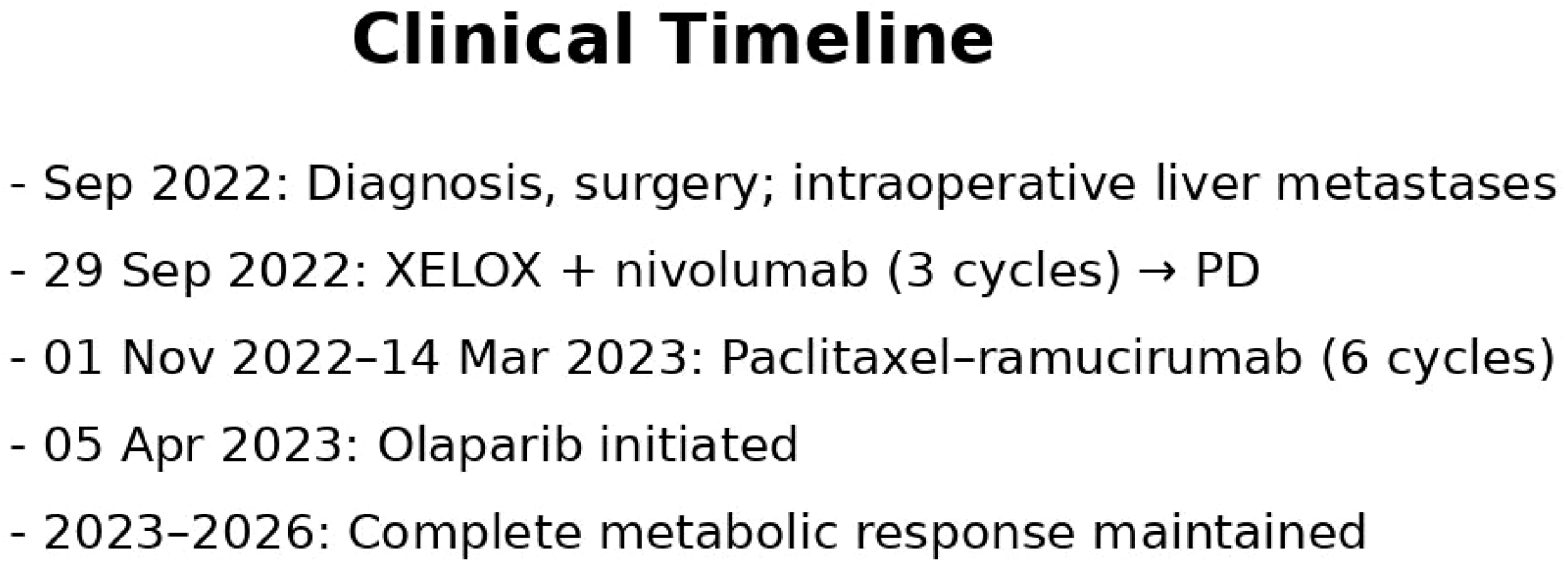
Clinical timeline of the patient from diagnosis to complete metabolic response under olaparib therapy.

A 65-year-old male presented in September 2022 with nonspecific upper gastrointestinal symptoms (**Figure 1**), including dyspepsia and early satiety. His medical history was notable for chronic cardiac arrhythmia requiring permanent pacemaker implantation approximately three years prior. He had no history of smoking or excessive alcohol consumption. At the time of diagnosis, there was no known personal or family history of malignancy or hereditary cancer syndromes. Approximately two years after the patient’s diagnosis, his daughter was diagnosed with breast cancer.

Initial staging with contrast-enhanced computed tomography and FDG PET-CT demonstrated a hypermetabolic gastric primary lesion without evidence of distant organ metastasis. Based on these findings, the patient underwent gastrectomy with curative intent. Intraoperatively, three nodular lesions suspicious for metastases were identified in the liver and were resected. Histopathological evaluation confirmed gastric adenocarcinoma with metastatic involvement of the liver and lymph node metastasis along the greater curvature. Surgical margins were negative, and the final pathological staging was pT3N1M1, consistent with stage IV disease.

### Diagnostic assessment

**Figure 2:**
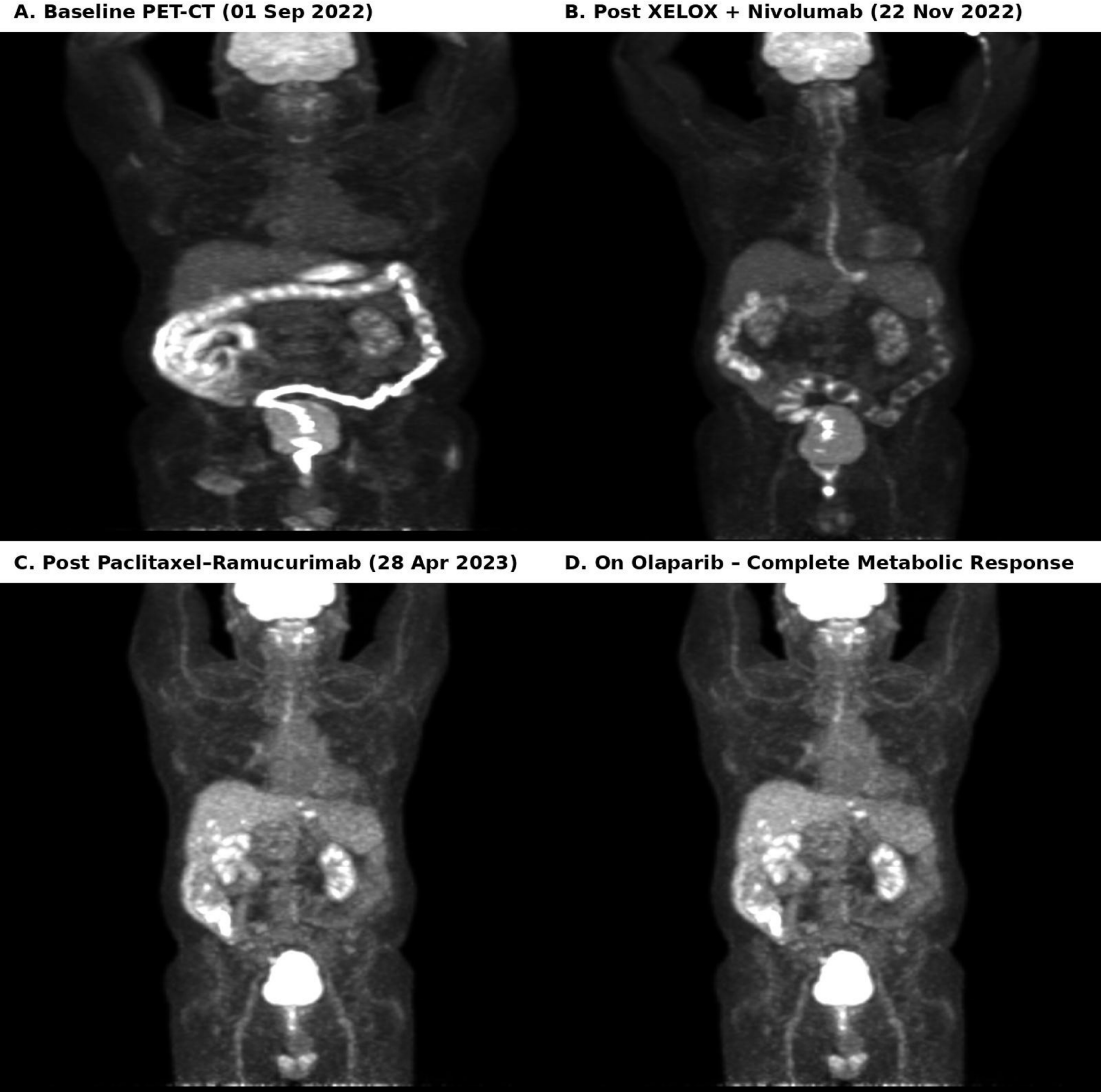
Serial FDG PET-CT images demonstrating disease progression after chemo-immunotherapy and subsequent complete metabolic response following olaparib treatment.

Radiologic disease assessment throughout the clinical course relied on serial FDG PET-CT imaging (**Figure 2**). PET-CT performed after three cycles of first-line XELOX plus nivolumab in November 2022 demonstrated progressive disease (2). A subsequent PET-CT obtained in April 2023 following completion of paclitaxel-ramucirumab therapy confirmed persistent metabolically active disease (6).

**Figure 3:**
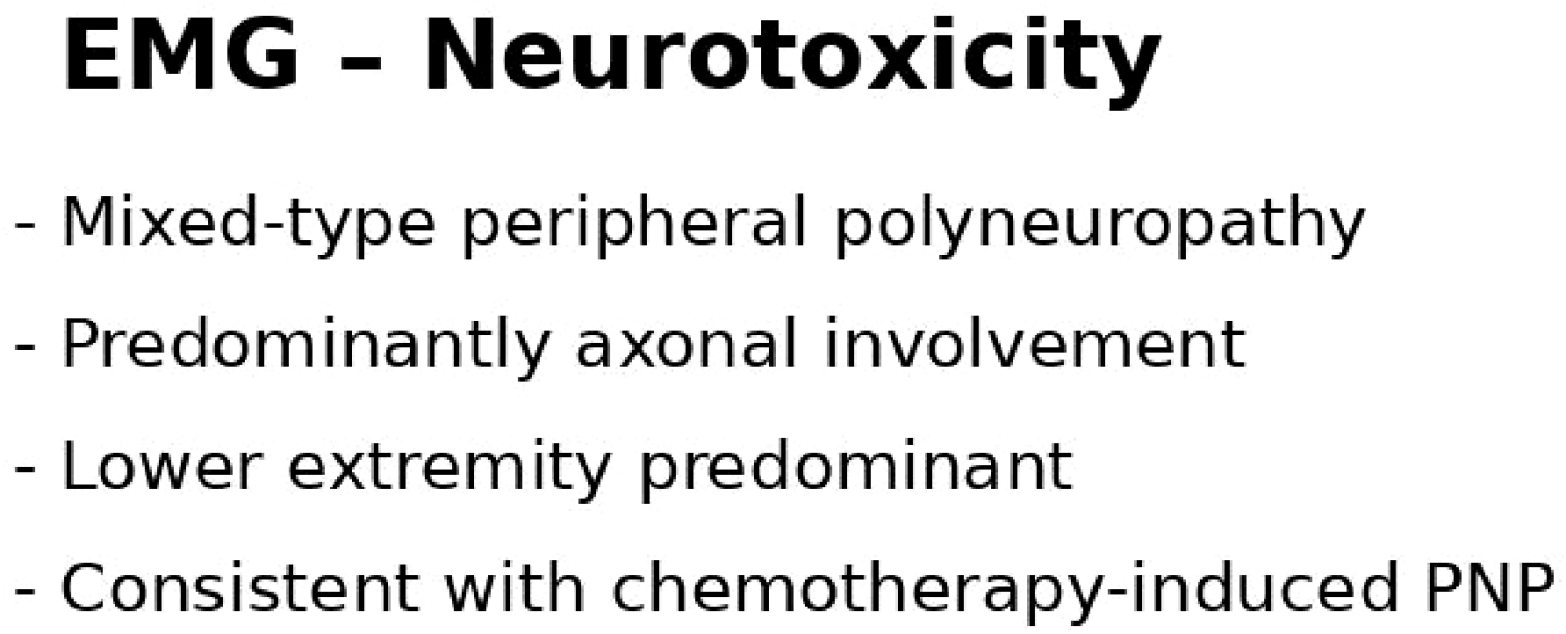
Electrophysiological findings consistent with chemotherapy-induced peripheral neuropathy.

Given progressive sensory complaints (**Figure 3**), nerve conduction studies were performed. Electrophysiological evaluation revealed a mixed-type peripheral polyneuropathy with predominant axonal involvement, more pronounced in the lower extremities, consistent with chemotherapy-induced peripheral neuropathy.

### Molecular profiling

**Figure 4:**
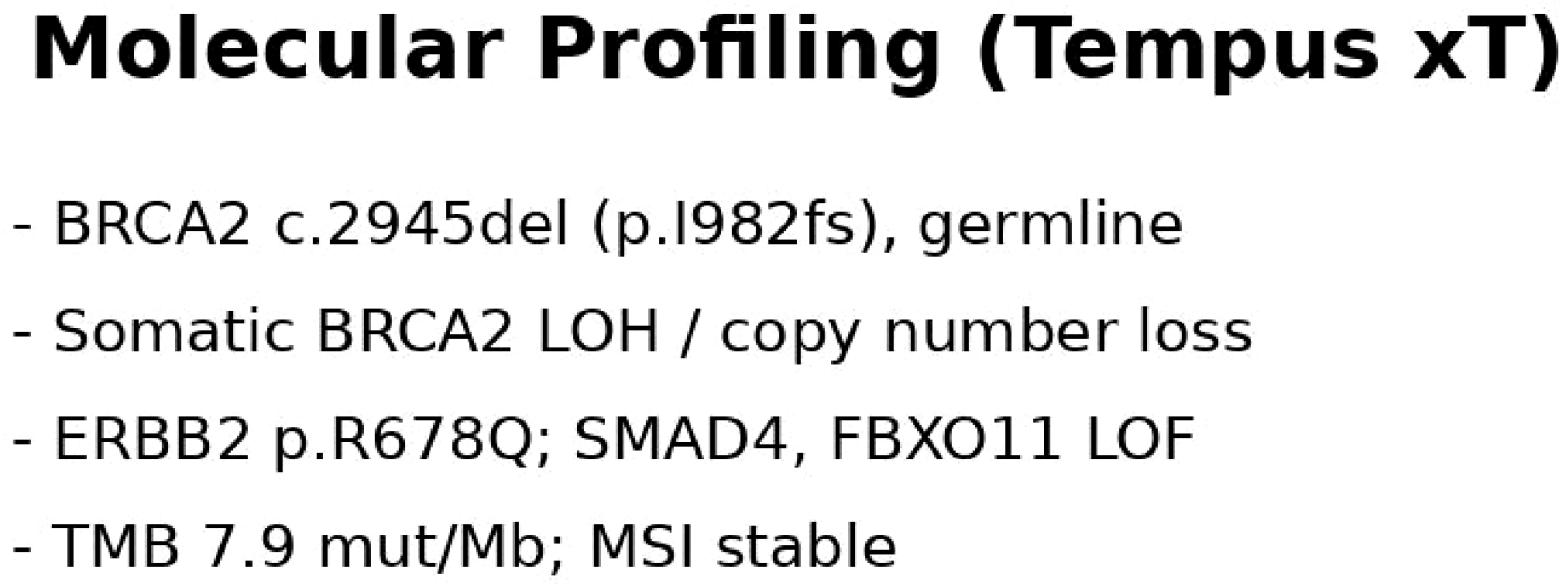
Summary of molecular profiling results showing BRCA2 mutation and associated genomic alterations.

Comprehensive genomic profiling was performed using the Tempus xT assay (648-gene panel) on a metastatic lymph node specimen, with matched peripheral blood as the normal control (**Figure 4**). Tumor cellularity was approximately 70%. The analysis identified a pathogenic germline BRCA2 frameshift mutation (c.2945del, p.I982fs), consistent with loss-of-function. In addition, somatic copy number loss and loss of heterozygosity of BRCA2 were detected, supporting a BRCA2-driven tumor biology (4, 7).

Additional somatic alterations included an activating ERBB2 (HER2) missense mutation (p.R678Q), as well as loss-of-function mutations in SMAD4 and FBXO11. Tumor mutational burden was in the 81st percentile (7.9 mutations/Mb), and microsatellite stable (MSS). No targetable gene fusions were identified.

Based on the presence of a pathogenic germline BRCA2 mutation with somatic loss of heterozygosity and prior platinum exposure, the tumor was considered homologous recombination repair deficient, providing a strong biological rationale for PARP inhibitor therapy (3, 4, 7).

### Therapeutic intervention

In light of disease progression on standard therapies, intolerance to further cytotoxic chemotherapy, and molecular findings consistent with homologous recombination repair deficiency, off-label olaparib therapy was initiated on 5 April 2023 at a standard dose of 300 mg twice daily (3). The patient was closely monitored for adverse events, treatment adherence, and laboratory abnormalities. No dose reductions or treatment interruptions were required.

### Follow-up and outcomes

Following initiation of olaparib therapy, serial FDG PET-CT imaging demonstrated marked metabolic regression, culminating in a complete metabolic response. The patient has remained on continuous olaparib therapy for more than three years with sustained disease control.

At the most recent follow-up, the patient’s performance status had improved to ECOG 1. Chemotherapy-induced peripheral neuropathy persisted at grade 2 severity but remained stable and was managed with pregabalin 75 mg twice daily. No clinically significant olaparib-related adverse events were observed.

### Patient perspective

The patient reported a substantial improvement in quality of life after discontinuation of cytotoxic chemotherapy and initiation of olaparib therapy. He described olaparib as more tolerable than previous treatments and expressed satisfaction with the durable disease control achieved.

## Discussion

This case report describes an exceptional and durable complete metabolic response to olaparib in a patient with BRCA2-driven metastatic gastric adenocarcinoma following progression on platinum-based chemotherapy combined with immune checkpoint inhibition and subsequent anti-angiogenic chemotherapy. The magnitude and durability of response observed in this heavily pretreated patient underscore the potential clinical relevance of homologous recombination repair deficiency–guided treatment strategies in gastric cancer.

Although BRCA1/2 alterations are well-established predictive biomarkers for PARP inhibitor sensitivity in breast, ovarian, pancreatic, and prostate cancers, their role in gastric cancer remains poorly defined. Previous clinical trials evaluating PARP inhibitors in unselected gastric cancer populations have failed to demonstrate meaningful clinical benefit, likely due to the absence of biomarker-based patient selection (5). In contrast, our case supports the hypothesis that a molecularly defined subset of gastric cancers harboring deleterious BRCA2 alterations with biallelic inactivation may exhibit marked sensitivity to PARP inhibition.

The comprehensive molecular profiling performed in this patient revealed a pathogenic germline BRCA2 frameshift mutation accompanied by somatic loss of heterozygosity, providing a strong biological rationale for synthetic lethality through PARP inhibition (3, 4, 7). Prior exposure and progression on platinum-based chemotherapy further support the presence of an underlying homologous recombination repair–deficient tumor biology. In addition, the durable response observed despite intermediate tumor mutational burden and microsatellite-stable status suggests that PARP inhibitor efficacy in this context is independent of classical immunotherapy biomarkers.

From a clinical perspective, this case highlights several important considerations. First, comprehensive genomic profiling may identify actionable vulnerabilities even in heavily pretreated gastric cancer patients with limited standard treatment options. Second, olaparib was well tolerated in this patient, with no need for dose reductions or interruptions, allowing prolonged treatment duration and sustained disease control. Third, objective confirmation of chemotherapy-induced peripheral neuropathy by electrophysiological studies supported the clinical decision to discontinue further cytotoxic therapy and pursue a targeted approach.

Several limitations warrant consideration. This report represents a single patient observation and therefore cannot establish causality or generalizability. Serial functional homologous recombination deficiency assays were not performed during treatment, and alternative biological mechanisms contributing to the observed response cannot be fully excluded. Nevertheless, the consistency between molecular findings, treatment response, and long-term disease control renders this case hypothesis-generating and clinically informative.

Future prospective studies incorporating comprehensive genomic profiling and functional HRD assessment are needed to better define the subset of gastric cancer patients most likely to benefit from PARP inhibitor therapy.

## Conclusion

This case demonstrates a durable complete metabolic response to olaparib in a patient with BRCA2-driven metastatic gastric adenocarcinoma following failure of chemo-immunotherapy and anti-angiogenic treatment. Comprehensive molecular profiling played a pivotal role in identifying a therapeutically actionable vulnerability and guiding treatment selection. These findings support further investigation of PARP inhibitors in biomarker-selected gastric cancer populations and highlight the importance of precision oncology approaches in advanced disease.

## Data Availability

The original contributions presented in the study are included in the article/supplementary material. Further inquiries can be directed to the corresponding author.
